# Nonlinear Dual Reconstruction of SPECT Activity and Attenuation Images

**DOI:** 10.1371/journal.pone.0106951

**Published:** 2014-09-16

**Authors:** Huafeng Liu, Min Guo, Zhenghui Hu, Pengcheng Shi, Hongjie Hu

**Affiliations:** 1 State Key Laboratory of Modern Optical Instrumentation, Department of Optical Engineering, Zhejiang University, Hangzhou, China; 2 B. Thomas Golisano College of Computing and Information Sciences, Rochester Institute of Technology, Rochester, New York, United States of America; 3 Department of Radiology, Sir Run Run Shaw Hospital, College of Medicine, Zhejiang University, Hangzhou, China; Washington State University, United States of America

## Abstract

In single photon emission computed tomography (SPECT), accurate attenuation maps are needed to perform essential attenuation compensation for high quality radioactivity estimation. Formulating the SPECT activity and attenuation reconstruction tasks as coupled signal estimation and system parameter identification problems, where the activity distribution and the attenuation parameter are treated as random variables with known prior statistics, we present a nonlinear dual reconstruction scheme based on the unscented Kalman filtering (UKF) principles. In this effort, the dynamic changes of the organ radioactivity distribution are described through state space evolution equations, while the photon-counting SPECT projection data are measured through the observation equations. Activity distribution is then estimated with sub-optimal fixed attenuation parameters, followed by attenuation map reconstruction given these activity estimates. Such coupled estimation processes are iteratively repeated as necessary until convergence. The results obtained from Monte Carlo simulated data, physical phantom, and real SPECT scans demonstrate the improved performance of the proposed method both from visual inspection of the images and a quantitative evaluation, compared to the widely used EM-ML algorithms. The dual estimation framework has the potential to be useful for estimating the attenuation map from emission data only and thus benefit the radioactivity reconstruction.

## Introduction

Single photon emission computed tomography (SPECT) has become an indispensable tool in clinical trials and medical practice. Attenuation correction in SPECT has significant values to better understand the physiological processes associated with the disease (i.e. cancers, heart diseases) and to provide improvement in patient diagnosis and treatment. However, even with ample efforts devoted to the development of various attenuation estimation and compensation techniques, it remains one of the key open issues in SPECT imaging [1]–[11].

Tissue attenuation map is usually estimated based on transmission data by scanning the patient with a rotating external radionuclide source [3], [5], [6], [12], or obtained from X-ray computed tomography (CT) system [10], [11], [13]–[16]. However, transmission based attenuation correction clearly increases the patient's dose, and requires maintaining additional radioactive sources. Further, if multiple imaging sessions are needed, it may be difficult for some patients to tolerate for a longer scan at one time, and leads to co-registration problem in emission image reconstruction, especially for deformed tissues and organs. In addition to the added equipment cost and the well-known beam hardening problem, similar registration issue exists for CT attenuation data.

It has been the goal of many recent research efforts to simultaneously estimate the activity and attenuation distributions from emission data. Iterative statistical methods have been extensively studied, with the main incentive being that they explicitly take into account the specific SPECT data statistics. Some of the most notable works include the use of differential attenuation method [17], gradient ascent [18], Tikhonov regularization [19], and expectation maximization (EM) [20]–[23]. With the SPECT imaging and measurement processes in state space representation, a recent work has adopted the extended Kalman filtering (EKF) procedures to linearize the augmented state representation to provide the joint estimates in the minimum-mean-square-error sense [24]. Such EKF based framework, however, has several potential drawbacks. First, the derivation of the Jacobian matrices, the linear approximations to the nonlinear functions, often leads to filter instability. Furthermore, it has been shown that estimation bias may originate from a coupling between the state variables and the model parameters, which suggests that if the system parameters can be separated from the system state variables, the precision of the estimation could be improved.

Following the same spirit while addressing the limitations, we present a robust unscented Kalman filter framework for the joint estimation of SPECT activity and attenuation parameters. Instead of linearizing using the Jacobian matrices and thus overcoming the associated shortcomings, our effort deals with the nonlinear estimation process by using a deterministic sampling approach to capture the mean and covariance estimates [25]. In addition, two iteratively coupled filters are used to sequentially estimate the activity variables (with fixed attenuation estimates from the last iteration) and the attenuation map (with fixed activity values from the previous estimation). Furthermore, such framework can explicitly recognize the uncertainties in the measurement data and the model structure, thus has the potential to produce more robust reconstructions.

## Materials and Methods

### SPECT Emission Scan Model

In SPECT imaging, once radio-tracers are injected into the subjects, they are delivered to the tissues/organs by the blood flow and participate in the related physiologic/metabolic processes. The general mathematical model for the emission scan can be stated as 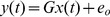
(1)where column vector 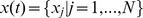 is the radiopharmaceutical concentration of the object, 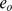 is the background noises (e.g. scatter events), and 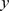 is the emission measurement data that is acquired by a rotating detector head around the patient at each angle and represented lexicographically as a column vector 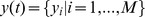. Here, 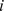 indicates different projection defined by rotating angle and different detector bin, and 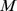 is the total number of projections. 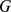 represents the emission system matrix including attenuation effects, and its element indicates the probability that a photon emitted from pixel gets detected in a specific detector bin.

Let the attenuation map be given by column vector 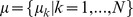, we may explicitly account for the attenuation effects in the system matrix 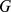 by factoring it as 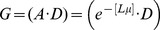
(2)


Here, The symbol ‘

’ means the dot product of two matrices. 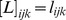 represents the length of the ray through voxel 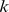 with respect to a photon emitted from voxel 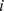 being detected in projection bin 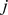. The photon survival probability considering attenuation can now be represented by a matrix 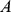 with elements 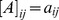 as 
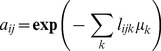
(3)


The term 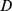, with elements 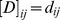, is the photon-detection probability without taking into account the attenuation effects.

The mathematical model of the SPECT emission measurement (Eqn. (1)) can now be rewritten as 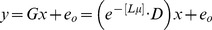
(4)or in discrete form 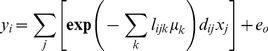
(5)


In the following sections, we will present the dual estimation method which works by alternating between using the UKF state-filter to estimate the radioactivity based on fixed attenuation parameters, and the UKF parameter-filter to estimate the attenuation coefficients given previously estimated activity states. Although two separate state-space representations are constructed for the activity and attenuation estimation problems, both of them use the same emission scan model (Eqn. (4) or (5)) as the measurement equation.

### Activity Estimation: UKF State Filter

#### State Space Representation for Radioactivity

In emission tomography, the goal is to reconstruct the radioactivity distribution 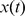 from the measurement data 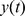. The system equation of the SPECT imaging system, which describes the radioactivity evolution of the pixels, can be written in the form of 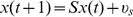
(6)with initial activity 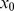 and system noise 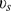 that accounts for the statistical uncertainty of the imaging model. In general, Eqn. (6) represents the dynamic changes of the state variable 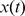, and it reduces to the conventional static reconstruction problem when the transition matrix 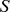 is an identity. The associated measurement equation, which describes the observations provided by the imaging data 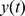, is given by Eqn. (4). And now we have the following state space representation for radioactivity distribution: 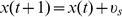
(7)

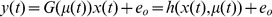
(8)where 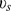 and 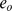, with covariance matrices 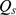 and 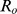, model the uncertainties of the imaging system and the measurement data respectively.

Two important observations on the nonlinear SPECT imaging system represented by Eqns. (7) and (8). First, since the noises in emission sinogram are typically Poisson distributed, it is difficult to perform standard estimation on such non-Gaussian system. By applying the Anscombe transformation [26], however, the Poisson noise could be converted into a Gaussian one, and various Kalman filtering techniques become viable options. Secondly, the EKF, probably the most widely used estimation algorithm for nonlinear systems, has several drawbacks. EKF requires the system is almost linear on the time scale of the updates, and is difficult to implement and to set proper parameters due to the cross-talk between state and parameter. To overcome such limitations, we have adopted the unscented transformation (UT) to accurately propagate mean and covariance information through nonlinear transformations [25], with little additional computational cost.

#### UKF State-Filter

For the SPECT imaging system given by Eqns. (7) and (8), the purpose of the UKF state-filtering is to search for the optimal estimates of radio-tracer variable 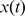, given fixed attenuation values 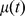. Since detailed discussion of the unscented Kalman filtering is certainly beyond the scope of this paper [25], we present here a more algorithmic description while ignoring some theoretical considerations.

The unscented transformation is a method for calculating the statistics of a random variable. Given a random variable 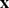 (dimension L) with mean 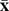 and covariance 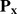 through a nonlinear function 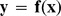. To compute the statistics of 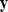, we form a sigma point matrix 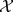 and their weights according to the following: 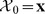


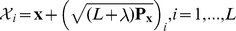


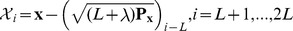


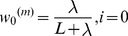
(9)

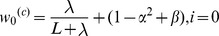


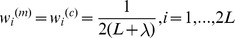
where 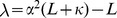. The parameter 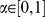 determines the spread of the sigma points, 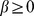 is used to incorporate any prior knowledge about the distribution of 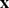, and 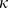 is a scaling parameter and is often set to be zero. Once having the above definitions, the UKF state-filter is initialized with 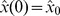 and covariance matrix 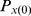, the state estimates and their error covariance matrices are computed sequentially until convergence:

1. Calculate the sigma point weights as in (9);
2. Project the state variable 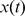 ahead: 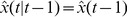
(10)
3. Project the error covariance 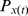 ahead: 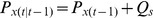
(11)
4. Calculate the sigma points as defined in Eqn. (9): 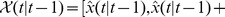


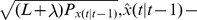


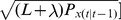
(12)
5. Filtering of the measurement equations: 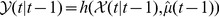
(13)

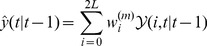
(14)

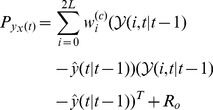
(15)
6. Compute the Kalman Gain: 
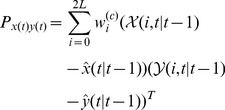
(16)

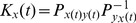
(17)
7. Update the estimate with the measurement: 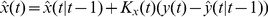
(18)
8. Update the error covariance: 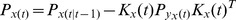
(19)Here, the previously stored information in the prediction step is combined with the new information coming from the next measurement 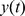 and the Kalman gain matrix to refine 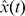 and 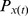 in the correction step. The covariance of the measurement error 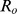 and system error 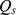 is assumed to be known and set to time-invariant.

### Attenuation Estimation: UKF Parameter Filter

Once the UKF state-filter converges, it is followed by the estimation of a coupled UKF parameter-filter aiming to recover the attenuation map of the object being imaged, given the estimated radioactivity map. The system equations for the parameter-filter are 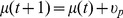
(20)

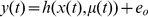
(21)


Here, 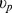 is the process noise with covariance matrix 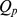, and we assume that the attenuation parameter vector 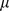 is temporally constant. Initializing the unscented parameter filter with 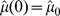 and covariance matrix 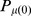, the parameter-filter follows similar recursion steps as the state-filter (Eqns. (9)–(17)) until convergence, given the sigma point calculation scheme of Eqn. 9.

The coupled radioactivity state and attenuation parameter estimation processes are iteratively repeated as necessary, until stable results are achieved. The final optimal estimates then become the reconstructed SPECT activity and attenuation maps.

## Results

### Validation with Synthetic Data

**Figure 1:**
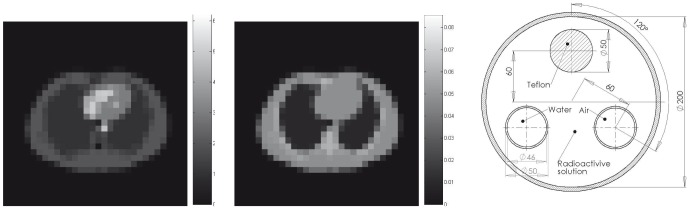
Simulated and physical phantom used in the experiments. From left to right: activity (*left*) and attenuation (*middle*) distributions of simulated Zubal phantom, physical imaging phantom with three different material rods inside. (*right*)

Synthetic Zubal phantom is used to quantitatively evaluate the accuracy and robustness of the framework, where a simplified human thorax with two lungs is represented by 32
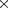
32 attenuation and activity (Fig. 1). Specifically, while both lungs have average attenuation coefficient of 0.04/cm, attenuation of the left lung is nonuniform but that of the right lung is uniform. SPECT projection data have been generated for a parallel beam geometry and 90 views uniformly spaced over 360 degrees. To generate realistic data, simulations in our study are performed using toolbox GATE [27], which can provide a relatively accurate reference for the assessment of new image reconstruction algorithms. And we have performed two studies, one with mean activities of 50 counts/pixel (low count), and the other with 200 counts/pixel (high count).

For these two sets of synthetic projection data, the radioactivity maps are reconstructed using four reconstruction methods: FBP [28], EM-ML [29], EKF [24], and dual UKF (The attenuation maps are also reconstructed for the EKF and UKF methods). For the UKF framework, the state (activity map) is estimated by the UKF state-filter, where, for the first iteration, the attenuation parameters come from the initial guess. Otherwise, we use the values estimated in the last iteration from the UKF parameter-filter. Consequently, these state estimates are used by the UKF parameter-filter to recover the attenuation coefficients. This process is executed iteratively until it meets the convergence criterion, which is defined using two consecutive normalized errors 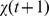 and 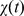 through 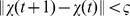 with 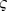 being a small constant, and 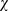 defines the normalized error between the estimated and the exact value (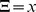 for the state-filter process, 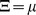 for the parameter-filter process) with 
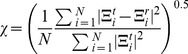
(22)where 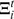 is the estimated value, 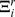 is the corresponding true value, and 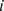 indicates the pixel.

A detailed statistical analysis on the estimation results against the ground truth phantom map is performed. Let 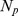 and 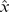 be the total number of pixels in the region of interest(ROI), e.g. body anatomy, and the final reconstruction results respectively, and 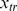 be the ground truth, we have the following error definitions: 
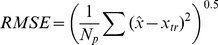
(23)


**Figure 2:**
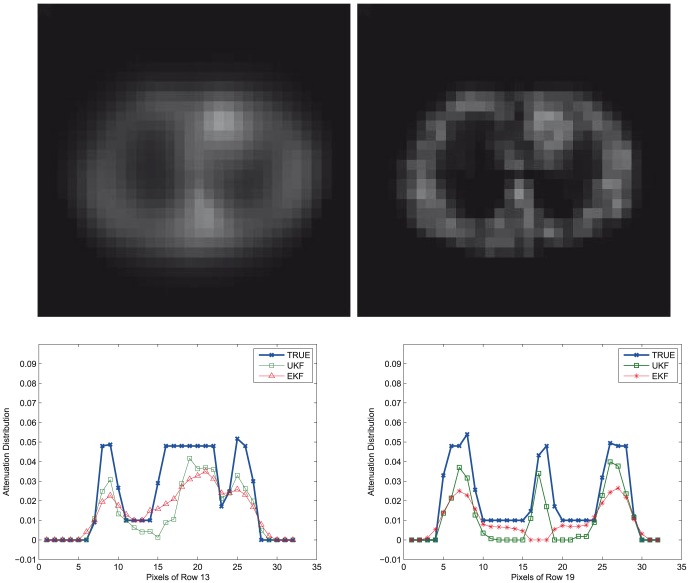
Synthetic Data. Top: Attenuation maps recovered by the EKF (*left*) and dual UKF (*right*) frameworks for low count measurements. Bottom: Horizontal profiles along the 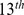 (*left*) and 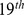 (*right*) rows of the recovered attenuation maps.

The results for the reconstruction of the attenuation maps are shown in Fig. 2 for the low count case. Visually, the UKF-reconstructed attenuation image clearly shows all relevant anatomical structures, where the lungs are easily seen with clear shape and size. The RMSE values in ROI of body anatomy using the UKF method are 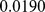 (low counts) and 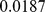 (high counts), which, though not as impressive, are still somewhat smaller than the corresponding values of 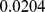 (low counts) and 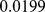 (high counts) obtained by the EKF method (note that the true average attenuation coefficient is 0.04). The worse performance of the EKF strategy could be caused by the first-order Taylor approximations of state transition that provided insufficiently accurate representations.

**Figure 3:**
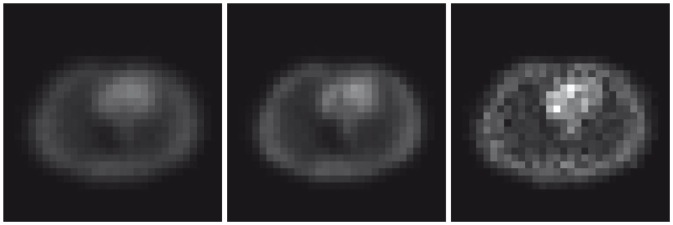
Synthetic Data. From left to right: activity maps recovered by FBP, EM-ML, and UKF methods for high count measurements.

**Figure 4:**
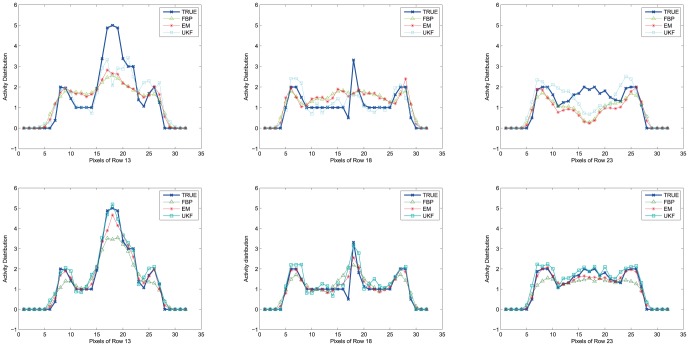
Synthetic Data. Top: Horizontal profiles along the 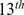 (*left*), 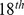 (*middle*), and 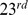 (*right*) rows of the recovered activity maps for low count measurements. Bottom: Horizontal profiles along the 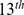 (*left*), 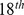 (*middle*), and 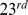 (*right*) rows of the recovered activity maps for high count measurements.

The results for the reconstruction of the activity maps are presented in Fig. 3, and the quantitative tabulation of the reconstruction accuracy is in Table 1. These figures and results illustrate that traditional EM-ML and FBP methods, with unknown attenuation map settings, produce some noticeable errors. The dual UKF estimation framework, on the other hand, consistently yields the best quality radioactivity estimates for both high and low count data. Same conclusion can be drawn from the visual examples of the selected horizontal profiles, as shown in Fig. 4.

**Table 1:** RMSE values of estimated activity maps for the synthetic data.

	FBP	EM	EKF	UKF
low count measurement	0.6724	0.5469	0.4152	0.3660
high count measurement	0.5252	0.3933	0.3710	0.3575

### Reconstruction from Physical Phantom Scanning Data

**Figure 5:**
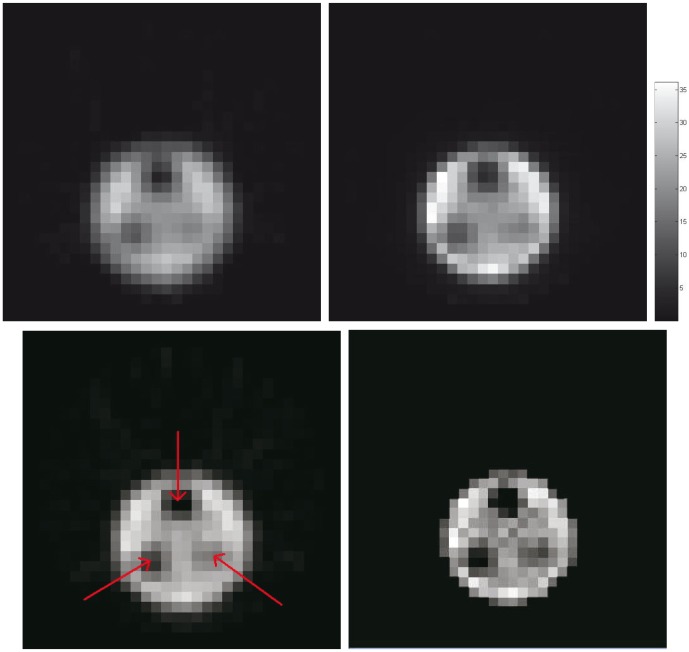
Reconstructed activity map of the physical phantom by FBP (*top left*), EM-ML (*top right*), EKF (*bottom left*) and UKF(*bottom right*) (arrows indicate cold areas), and the associated color scale.

**Figure 6:**
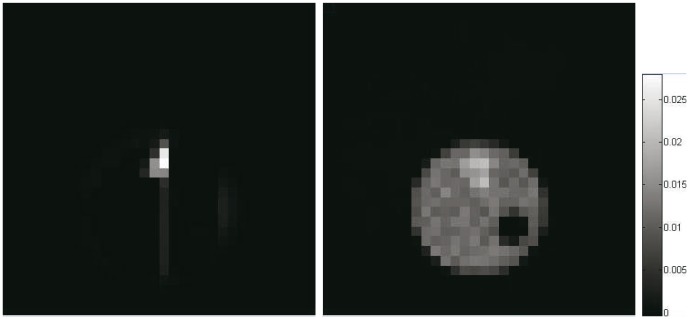
Reconstructed attenuation map of the physical phantom by EKF (*left*) and UKF (*right*), and the associate color scale.

The second data set used for validation has been on a real cylinder phantom. The dimension of the phantom is 200 mm (diameter) 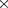 290 mm (depth). A Teflon rod and two hollow PMMA cylinders with diameters of 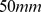 are inserted in the phantom's volume, as shown in Fig. 1. The phantom is filled with 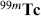 concentration with a total radioactivity of 20mCi (100kBq/cc) and the two hollow cylinder rods are filled with air and pure water respectively. The phantom was scanned with a Siemens ECam^*duet*^ ECT scanner by two detector head rotating at total 64 angle position around 180 degree and the acquiring time at each position is 30 seconds. The final sinogram data has 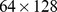 projections for each slice. Once again, FBP, EM-ML, EKF and dual UKF strategies have been used to reconstruct activity maps from the measurement data, as shown in Fig. 5. Visually, it is evident that the UKF method produces the best reconstruction results, especially for the three cold areas. The results of EKF and UKF reconstructed attenuation map are shown in Fig. 6, with RMSE values of 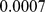 for UKF and 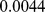 for EKF.

### Reconstruction from Real Patient Scanning Data

**Figure 7:**
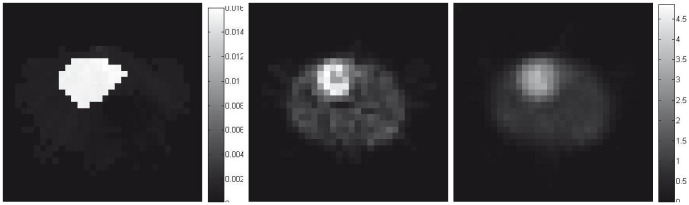
Reconstructed attenuation (*left*, by UKF) and activity maps of the patient by UKF (*middle*) and FBP (*right*), and the associated color scales.

The dual reconstruction strategy has also been evaluated on clinical studies, where the patients are undergoing 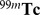 sestamibi stress tests. Using a Siemens ECam^*duet*^ scanner, all projections are acquired over 120 angles covering a circular 360 degrees acquisition orbit in a continuous step-and-shoot mode. With an acquisition time of 16s/frame, the total photon counts for each slice are 148761. The dual reconstruction of activity and attenuation maps are shown in Fig. 7, and the clinically standard FBP reconstruction (without attenuation correction) is also shown for comparison. Since the transmission data is not available from this imaging site, we can only make qualitative visual inspection of the images. It is quite clear, however, that the estimated attenuation map agrees with general knowledge of the imaged area, and the UKF estimated activity map exhibits improved contrast between heart and soft tissue.

There is usually a considerable increase in computation for improved performance. The computational load increases when moving from the EM to the UKF. However, as the UKF gives a better approximation in time update step, the UKF estimate is able to converge quite faster comparing to the EM. Furthermore, this proposed approach runs efficiently on graphics processing units(GPUs) since large amounts of computations are done in matrix forms. Further investigations on the implementation with GPUs are underway.

## Conclusions

A dual UKF strategy has been derived for joint reconstruction of the attenuation map and activity distribution solely from SPECT emission sinograms. Constructing the state transition of the activity distribution through state space evolution equations and the photon-counting measurements through observation equations, we rely on the unscented Kalman filter principles to first generate estimates of activity maps with sub-optimal attenuation parameter estimates, and then recover the attenuation maps given these activity estimates. These coupled iterative steps are repeated as necessary until convergence. Simulated and physical phantoms, as well as real patient data, are used to evaluate the proposed strategy.

## References

[1] King MA, Tsui BM, Pan T (1995) Attenuation compensation for cardiac single-photon emission computed tomography imaging: Part 1. Impact of attenuation and methods of estimating attenuation maps. J Nucl Cardiol 2: 513–524. 9420834 10.1016/s1071-3581(05)80044-3

[2] Welch A, Clack R, Natterer F, Gullberg G (1997) Toward accurate attenuation correction in SPECT without transmission measurements. IEEE Trans Med Imag 16: 532–541. 10.1109/42.640743 9368109

[3] Zaidi H, Hasegawa B (2003) Determination of the attenuation map in emission tomography. J Nucl Med 44: 291–315. 12571222

[4] Ficaro E, Corbett J (2004) Advances in quantitative perfusion SPECT imaging. J Nucl Cardiol 11: 62–70. 14752474 10.1016/j.nuclcard.2003.10.007

[5] Celler A, Dixon KL, Chang Z, Blinder S, Powe J, et al (2005) Problems created in attenuation-corrected SPECT images by artifacts in attenuation maps: A simulation study. J Nucl Med 46: 335–343. 15695795

[6] Feng B, Fessler JA, King MA (2006) Incorporation of system resolution compensation (RC) in the ordered-subset transmission (OSTR) algorithm for transmission imaging in SPECT. IEEE Trans Med Imag 25: 941–949. 10.1109/tmi.2006.876151 16827494

[7] Nunez M, Prakash V, Vila R, Mut F, Alonso O, et al (2009) Aattenuation correction for lung SPECT: evidence of need and validation of an attenuation map derived from the emission data. European Journal of Nuclear Medicine and Molecular Imaging 36: 1076–1089. 19238381 10.1007/s00259-009-1090-4

[8] Cuocolo A (2011) Attenuation correction for myocardial perfusion spect imaging: still a controversial issue. European journal of nuclear medicine and molecular imaging 38: 1887–1889. 21874324 10.1007/s00259-011-1898-6

[9] Garcia EV (2007) Spect attenuation correction: an essential tool to realize nuclear cardiologys manifest destiny. Journal of nuclear cardiology 14: 16–24. 17276302 10.1016/j.nuclcard.2006.12.144

[10] Wu C, van Andel HAG, Laverman P, Boerman OC, Beekman FJ (2013) Effects of attenuation map accuracy on attenuation-corrected micro-spect images. EJNMMI research 3: 1–11. 23369630 10.1186/2191-219X-3-7 PMC3579699

[11] Ishii K, Hanaoka K, Okada M, Kumano S, Komeya Y, et al (2012) Impact of ct attenuation correction by spect/ct in brain perfusion images. Annals of nuclear medicine 26: 241–247. 22278350 10.1007/s12149-011-0567-y

[12] McGowan SE, Greaves CD, Evans S (2012) An investigation into truncation artefacts experienced in cardiac imaging using a dedicated cardiac spect gamma camera with transmission attenuation correction. Nuclear medicine communications 33: 1287–1291. 23027382 10.1097/MNM.0b013e328359db76

[13] Fleming JS (1989) A technique for using CT images in attenuation correction and quantification in SPECT. Nucl Med Commun 10: 83–97. 2786613 10.1097/00006231-198902000-00002

[14] Kalki K, Blankespoor SC, Brown JK, Hasegawa BH, et al (1997) Myocardial perfusion imaging with a combined x-ray CT and SPECT system. J Nucl Med 38: 1535–1540. 9379188

[15] Kinahan PE, Townsend DW, Beyer T, Sashin D (1998) Attenuation correction for a combined 3D PET/CT scanner. Med Phys 25: 2046–2053. 9800714 10.1118/1.598392

[16] Willowson K, Bailey D, Baldock C (2008) Quantitative SPECT reconstruction using CT-derived corrections. Phys Med Biol 53: 3099–3112. 18495976 10.1088/0031-9155/53/12/002

[17] Kaplan MS, Haynor RS, Vija H (1999) A differential attenuation method for simultaneous estimation of SPECT activity and attenuation distributions. IEEE Nucl Sci 46: 535–541. 10.1118/1.598747 10587214

[18] Nuyts J, Dupont P, Stroobants S, Benninck R, Mortelmans L, et al (1999) Simultaneous maximum a posteriori reconstruction of attenuation and activity distributions from emission sinograms. IEEE Trans Med Image 18: 393–403. 10.1109/42.774167 10416801

[19] Dicken V (1999) A new approach towards simultaneous activity and attenuation reconstruction in emission tomography. Inverse Problem 15: 931–960.

[20] Krol A, Bowsher JE, Manglos SH, Feiglin DH, Tomai MP, et al (2001) An EM algorithm for estimating SPECT emission and transmission parameters from emissions data only. IEEE Trans Med Imag 20: 218–232. 10.1109/42.918472 11341711

[21] Gourion D, Noll D, Gantet P, Celler A, Esquerre J (2002) Attenuation correction using SPECT emission data only. IEEE Trans on Nuclear Science 49: 2172–2179.

[22] Jha AK, Clarkson E, Kupinski MA, Barrett HH (2013) Joint reconstruction of activity and attenuation map using lm spect emission data. In: SPIE Medical Imaging. International Society for Optics and Photonics, pp. 86681W–86681W. 10.1117/12.2008111 PMC4519980 26236067

[23] Salomon A, Goedicke A, Aach T (2011) Attenuation corrected cardiac spect imaging using simultaneous reconstruction and a priori information. IEEE Transactions on Nuclear Science 58: 527–536.

[24] Tian Y, Liu HF, Shi P (2006) Simultaneous reconstruction of tissue attenuation and radioactivity maps in SPECT. MICCAI I: 397–404. 10.1007/11866565_49 17354915

[25] Julier SJ, Uhlmann JK (2004) Unscented filtering and nonlinear estimation. Proceedings of the IEEE Aerospace and Electronic Systems 92: 401–422.

[26] Anscombe FJ (1948) The transformation of poisson, binomial and negative-binomial data. Biometrika 35: 246–254.

[27] Jan S, Santin G, Strul D, Staelens S, Assie K, et al.. (2004) GATE: a simulation toolkit for PET and SPECT. Physics in Medicine and Biology : 4543–4561. 10.1088/0031-9155/49/19/007 PMC3267383 15552416

[28] Kak AC, Slaney M (2001) Principles of computerized tomographic imaging. Society for Industrial and Applied Mathematics.

[29] Shepp LA, Vardi Y (1982) Maximum likelihood reconstruction for emission tomography. IEEE Transactions on Medical Imaging 1: 113–122. 18238264 10.1109/TMI.1982.4307558

